# Romantic Attachment, Stress, and Cognitive Functioning in a Large Sample of Middle-Aged and Older Couples

**DOI:** 10.1093/geroni/igab046.1094

**Published:** 2021-12-17

**Authors:** Rebekka Weidmann, William Chopik

**Affiliations:** Michigan State University, East Lansing, Michigan, United States

## Abstract

Romantic relationships are a key factor contributing to health across the lifespan. Within this research line, attachment theory has been a useful framework to understand how relationships impact health. One primary health concern in late adulthood is reduced cognitive functioning: Alzheimer’s disease and related neurodegenerative disorders become increasingly prevalent with age affecting millions of people. Even though much research has identified various sociodemographic, medical, and behavioral risk factors, little knowledge exists on romantic attachment’s psychosocial role for cognitive decline. The purpose of this study was to examine the link between insecure attachment, stress, and cognitive functioning in a large sample of middle-aged and older couples. In particular, we wanted to investigate how insecure attachment is linked to both partners’ cognitive functioning and whether stress mediates these associations. To that aim, we used data of 1,043 romantic couples (Mage = 64.7 years; 38.5% same-sex couples) who reported on their attachment anxiety and avoidance, their stress levels, their cognitive decline, and their and their partners’ dementia symptoms. Couple members also participated in a memory performance task. The results suggest that anxiety is linked to participants’ cognitive decline, while avoidance was linked to partners’ cognitive decline and poorer memory performance. We also detected significant mediational effects for stress in the association between insecure attachment and cognitive functioning. We conclude that potentially malleable psychosocial factors, such as insecure attachment and stress, are important research objects when understanding cognitive functioning in middle and late adulthood.

